# BLOOD THICKER THAN WATER? STEPGAP IN INTERGENERATIONAL RELATIONSHIPS

**DOI:** 10.1093/geroni/igz038.2481

**Published:** 2019-11-08

**Authors:** Peter Öberg, Torbjorn Bildtgard

**Affiliations:** 1 University of Gävle, Gävle, Sweden; 2 Stockholm University, Stockholm, Sweden

## Abstract

The increasing prevalence of ageing stepfamilies and stepchildren’s potential to act as a source of support for older parents has prompted research about intergenerational cohesion in step-relationships. Previous research has hypothesized a qualitative gap (a step-gap) between step and biological relationships to the advantage of the latter. In this Swedish study we compare emotional closeness between older parents and adult children among parents (aged 66-79) who have had both biological and stepchildren, and children (aged X-Y) who have had both biological and stepparents. Qualitative interviews (n=24) of family histories including a hierarchical convoy model of family relationships were collected and analyzed. Results show that with few exceptions biological relationships are rated as emotionally closer than step-relationships, both by parents and adult children, supporting the step-gap hypothesis. While the older parents tend to deemphasize the importance of blood for their ratings, the adult children often emphasize the importance of blood. The difference is explained by a parental adherence to an ideology of equal treatment of children, while the adult children stress the importance of biology for their identity and belonging.

